# Antibody-Functionalized Carnauba Wax Nanoparticles to Target Breast Cancer Cells

**DOI:** 10.1021/acsabm.1c01090

**Published:** 2022-01-03

**Authors:** Banu Iyisan, Johanna Simon, Yuri Avlasevich, Stanislav Baluschev, Volker Mailaender, Katharina Landfester

**Affiliations:** † Max Planck Institute for Polymer Research, Ackermannweg 10, 55128 Mainz, Germany; ‡ Institute of Biomedical Engineering, Boğaziçi University, 34684 Çengelköy, Istanbul, Turkey; § Dermatology Clinic, University Medical Center of the Johannes Gutenberg-University Mainz, Langenbeckstraße 1, 55131 Mainz, Germany; ∥ Faculty of Physics, University of Sofia “Saint Kliment Ohridski”, James Bourchier 5, 1164 Sofia, Bulgaria

**Keywords:** lipid nanoparticles, functionalization, cancer targeting, nanomedicine, isothermal titration calorimetry

## Abstract

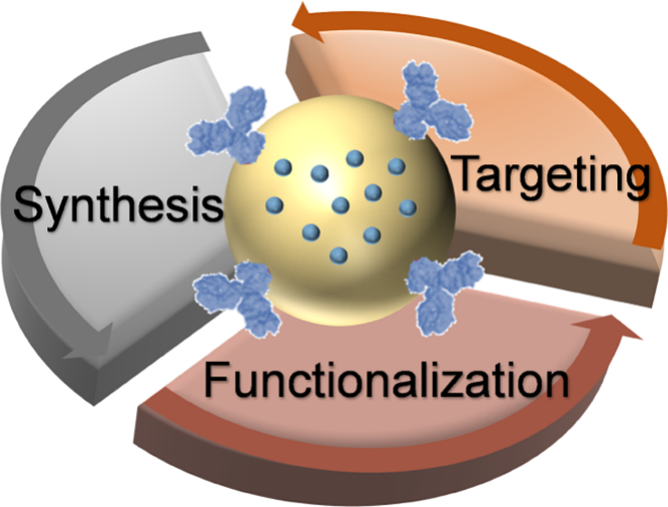

Development of safer nanomedicines for drug delivery applications requires immense efforts to improve clinical outcomes. Targeting a specific cell, biocompatibility and biodegradability are vital properties of a nanoparticle to fulfill the safety criteria in medical applications. Herein, we fabricate antibody-functionalized carnauba wax nanoparticles encapsulated a hydrophobic drug mimetic, which is potentially interesting for clinical use due to the inert and nontoxic properties of natural waxes. The nanoparticles are synthesized applying miniemulsion methods by solidifying molten wax droplets and further evaporating the solvent from the dispersion. The pH-selective adsorption of antibodies (IgG1, immunoglobulin G1, and CD340, an antihuman HER2 antibody) onto the nanoparticle surface is performed for practical and effective functionalization, which assists to overcome the complexity in chemical modification of carnauba wax. The adsorption behavior of the antibodies is studied using isothermal titration calorimetry (ITC), which gives thermodynamic parameters including the enthalpy, association constant, and stoichiometry of the functionalization process. Both antibodies exhibit strong binding at pH 2.7. The CD340-decorated wax nanoparticles show specific cell interaction toward BT474 breast cancer cells and retain the targeting function even after 6 months of storage period.

## Introduction

Multifunctional nanocarriers are vital tools to develop safer nanomedicines for drug delivery applications.^1^ One important safety criterion of nanomedicine is to target a specific cell for the selective delivery of drugs as therapeutic vehicles. In addition, the nanomedicine needs to be biocompatible, biodegradable, sufficiently circulate in the bloodstream, and have adequate cargo encapsulation capacity.^2,3^ To fulfill these key properties, the surface functionality, size, shape, colloidal stability, and composition of a nanocarrier should be precisely engineered.^4−6^ Yet, balancing all of these properties to design a safer and functional nanomedicine that does its task efficiently is a challenging topic.

Several studies to prepare safer and functional nanomedicines using distinct nanocarriers such as polymersomes,^7−9^ nanocapsules,^10−12^ nanospheres,^13,14^ and liposomes^15^ have been reported. Fabricating all of the mentioned carrier systems requires nontoxic ingredients to obtain clinical relevance.^16^ Thus, using a biocompatible material in nanocarrier formulations is of great importance. In relation to this, natural lipids like carnauba wax have received increased attention as a nanoparticle matrix due to their nontoxic and chemically inert properties.^17,18^ These lipids have been widely used in cosmetic,^19^ pharmaceutical,^20^ and food^21^ industries for oral or topical administration and can be a promising candidate to form biocompatible drug delivery systems. Carnauba wax is extracted from the leaves of a Brazilian palm tree (*Copernicia prunifera*) and has a high melting point (82–86 °C) and low water solubility. It is the hardest natural wax that contains predominantly aliphatic esters, diesters of cinnamic acid, and fatty alcohols.^21,22^ So far, there have been a few reports on carnauba wax nanoparticles for use in sunscreen formulations^23^ or for the oral delivery of antioxidants like rosmaniric acid.^24^ A mixture of beeswax and carnauba wax was also used to prepare nanoparticles for encapsulating ketoprofen, a drug having limited water solubility.^25^ To go one more step in this field, the advantage of the clinical relevance of carnauba wax-based nanoparticles should be combined with the recognition ability to target specific cells for their application as safer and smart drug carriers. However, at this point, a challenging question arises: how can we functionalize a wax nanoparticle that is difficult to alter chemically?

Functionalization of a nanoparticle surface to add targeting units like antibodies,^13^ sugars,^12^ and folic acids^8^ can be performed using covalent and noncovalent approaches. To use the covalent approaches, a suitable linker group such as amine moieties, carboxyl groups, or azido moieties is needed on the particle surface for covalently linking the targeting ligands/antibodies.^5^ Unlike polymeric materials, carnauba wax has no easily accessible functional units for further linker addition, which makes the covalent conjugations impractical. Besides, it is hard to process the wax material due to its low water solubility in mild conditions. Thus, noncovalent approaches like preadsorption of the targeting unit on a nanoparticle surface, as favored in the vaccine formation,^26^ can be a feasible method if a sufficiently stable binding is constructed. Recently, our group reported^27^ that pH-dependent preadsorption of antibodies onto polymeric nanoparticles made of polystyrene^28^ and hydroxyethyl starch (HES)^29^ results in functional targeting of nanocarriers toward monocyte-derived dendritic cells (moDCs). Therein, the targeting ability of preadsorbed antibodies was not disturbed by protein corona formation in plasma and showed better performance in comparison to covalently linked antibodies.^27^ This confirms that preserving the ligand or antibody specificity after modifying the nanoparticle surface is another challenge in the design of nanomedicines.^6^ Besides, strongly conjugated targeting units are required to avoid the desorption from the nanocarrier surface before the target cell is reached.^30^ A convenient tool to investigate the binding stability of the biomolecules on the nanoparticle surface is to utilize isothermal titration calorimetry (ITC), which enables interpreting thermodynamic adsorption parameters including the association constant (*K*_a_), the binding enthalpy (Δ*H*), and the stoichiometry (*n*).^31−33^

In this study, we demonstrate the development of clinically relevant antibody-functionalized carnauba wax nanoparticles to target human epidermal growth factor receptor 2 (HER2)-positive breast cancer cells. The previously described preadsorption methodology^27^ is applied for antibody decoration of carnauba wax nanoparticles, which provides a feasible and effective functionalization process. In this way, complexity in surface modification of nontoxic and chemically inert carnauba wax^21^ nanoparticles can be overcome. As a targeting unit, CD340, an antihuman HER2 antibody, is adsorbed onto the nanoparticle, whereas IgG1, immunoglobulin G1, is adsorbed as an isotype control to investigate the specific cell interaction for CD340-modified carnauba wax NPs (nanoparticles) when both of them were taken up by BT474 cells (HER2-positive cells). In addition, the encapsulation of a hydrophobic dye molecule as a model drug compound into the carnauba wax nanoparticles is also performed. Since the antibody-binding strength is an important parameter for the final nanomedicine performance, the thermodynamic adsorption parameters between the corresponding antibodies and the carnauba wax nanoparticles are monitored using ITC. This information is also supported by recording the physicochemical integrity and biological function of the antibody-bound nanoparticles for 6 months of storage period.

## Experimental Section

### Materials

**Figure 1:**
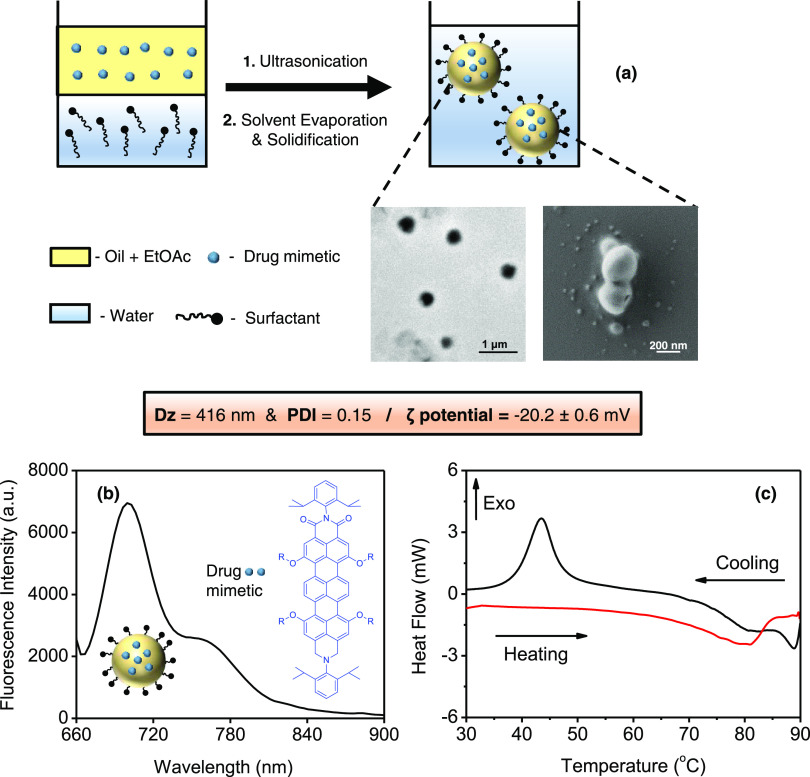
(a) Synthesis scheme of carnauba wax nanoparticles using miniemulsion techniques. TEM and SEM micrographs show the spherical morphology of the nanoparticles. Physicochemical characteristics include the size and ζ-potential values. (b) Fluorescence spectra of carnauba wax nanoparticles encapsulated with a drug mimetic (TDI, tracking molecule); λ_exc_ = 633 nm, R = (4-*tert*-octylphenoxy). (c) Differential scanning calorimetry (DSC) thermogram of carnauba wax nanoparticles showing the heating/cooling cycles.

Carnauba wax, toluene, and ethyl acetate were purchased from Acros Organics. Tween 20 (poly(ethylene glycol) sorbitan monolaurate), squalene, and the chemicals used for buffer preparation (glycine–HCl, MES hydrate, HEPES, NaHCO_3_, NaOH) were purchased from Sigma-Aldrich. Rice bran oil was purchased from TEA Natura (TEA Prodotti Naturali di Manzotti P., Italy). TDI dye (a drug mimetic, λ_exc_ = 630 nm, λ_em_ = 700 nm; the structure is given in Figure 1) was synthesized as reported previously.^34^ Mouse IgG1 (clone: MOPC-21) and antihuman CD340 (erbB2/HER2; clone: 24D2) were purchased from Biozol Diognostica Vertrieb GmbH and used as received. Sterile water (Braun Melsungen AG) was used for the experiments unless otherwise stated.

### Nanoparticle Synthesis

The nanoparticles were synthesized using the miniemulsion solvent evaporation technique. A mixture of rice bran oil (90 mg), squalene (90 mg), drug mimetic (TDI dye, 3 × 10^–7^ mol), and ethyl acetate (2 mL) was formed and added to the molten carnauba wax (120 mg) at 100 °C. The homogeneous mixture of the dispersed phase was mixed with a continuous phase comprising 5 mL of 1 vol % aqueous solution of Tween 20. Afterward, ultrasonication was performed for 4 min using a Branson W-450D Digital Sonifier at 90% amplitude (15 s pulse/5 s pause). The formed hot molten wax droplets were cooled down to room temperature and followed by the evaporation of ethyl acetate under reduced pressure for 5 h.

### Nanoparticle Characterization

Dynamic light scattering (DLS) measurements were performed at 25 °C using a Zetasizer Nano series instrument (Malvern Instruments, U.K.) equipped with a 633 nm He–Ne laser at a fixed scattering angle of 90° to determine the size of the nanoparticles as the intensity-average diameter (*z*_average_) values. The ζ-potential of the nanoparticles was determined by electrophoretic light scattering using a Zetasizer Nano Z instrument (Malvern Instruments, U.K.) in 1 mM potassium chloride solution at 25 °C. Fluorescence spectroscopy was performed using a Spex Fluorolog 3 spectrofluorometer. The nanoparticles were placed in a quartz cuvette and excited at 630 nm. The morphology of the nanoparticles was investigated by utilizing transmission electron microscopy (TEM) and scanning electron microscopy (SEM). TEM was performed using a JEOL 1400 transmission electron microscope (Jeol Ltd., Tokyo, Japan) operating at an acceleration voltage of 120 kV. Samples were prepared by dropping the diluted nanocapsule dispersions onto 300 mesh carbon-coated copper grids followed by the removal of excess water using a piece of filter paper to decrease artifacts caused by drying. SEM was performed using a Gemini 1530 (Carl Zeiss AG Oberkochem, Germany) operating at 0.35 kV. The samples were prepared by dropping diluted nanoparticles onto a silicon wafer and let them dry at ambient conditions before the observation. Differential scanning calorimetry (DSC) of nanoparticles (100 μL, 58.2 mg·mL^–1^) was performed using a Mettler Toledo DSC, operating at a 5 K/min heating rate (−140 to 90 °C) under nitrogen.

### Antibody Functionalization of Nanoparticles

About 2.5 mg of carnauba wax nanoparticles was incubated with 25 μg of mouse IgG1 or CD340 for 4 h at room temperature in 1 mL of buffer at various pH values. The buffer media were prepared from the following ingredients: pH 2.7 glycine–HCl (125 mM), pH 5.5 MES (50 mM), pH 6.1 MES (50 mM), pH 7.5 HEPES (50 mM), pH 9.5 NaHCO_3_ (50 mM), and pH 11 NaHCO_3_ (50 mM) and NaOH (100 mM). After the incubation, the nanoparticles were centrifuged for 1 h at 20 000*g* and room temperature three times to remove free antibodies and then resuspended in ultrapure water. Concentrations of the antibody-functionalized nanoparticles (*C*_NPCD340_= 1.8 mg·mL^–1^ and *C*_NPIgG1_ = 1.5 mg·mL^–1^) were determined by monitoring the fluorescence intensity using an Infinite M1000 plate reader (Tecan Group Ltd.). After nanoparticle synthesis, the solid content of unfunctionalized nanoparticles (62 mg·mL^–1^) was determined gravimetrically by freeze-drying the unfunctionalized nanoparticles. Afterward, nanoparticles were diluted with water up to a concentration of ∼100 to 3 μg·mL^–1^ and the fluorescence intensity was determined via the Infinite M1000 plate reader at 680/710 nm to obtain the calibration curve, as shown in Figure S5. By following this, the antibody-functionalized nanoparticles were diluted (1:100) and their fluorescence intensity was determined via the Infinite M1000 plate reader to calculate the concentration using the obtained calibration curve (Figure S5).

### Detection of Antibodies on the Nanoparticle Surface Using Flow Cytometry

The detection of antibodies on the nanoparticle surface was performed using our previously established method.^27^ The antibody-functionalized nanoparticles were incubated with secondary Alexa-Fluor 405-labeled antibodies for 30 min at room temperature in the dark. Afterward, the mixture was filled up with PBS (1 mL) and measured using an Attune Nxt flow cytometer (Thermo Fisher Scientific) equipped with four lasers (405, 488, 561, and 637 nm) and 16 channels (VL1–4, BL1–4, YL1–4, RL1–4). Next, the number of Alexa-Fluor 405-labeled nanoparticles was quantified in a histogram by reporting the amount (%) of Alexa-positive nanoparticles. Results are given as the average of triplicates.

### Isothermal Titration Calorimetry (ITC)

ITC measurements were performed using a NanoITC Low Volume (TA Instruments, Eschborn, Germany) with an effective cell volume of 170 μL. In each experiment, 50 μL of aqueous antibody solution (IgG1 or CD340, 30 μg) was titrated into 300 μL of carnauba wax nanoparticles (*c* = 1.78 × 10^–6^ mM) at a stirring rate of 250 rpm. For the sample preparation, the nanoparticles and antibodies were mixed with either MES buffer (50 mM, pH 6.1) or glycine–HCl (125 mM, pH 2.7) to regulate the pH at corresponding values as likely done in antibody adsorption experiments. The temperature was kept constant at 25 °C throughout the titration. As a reference, the same amount of antibody solution was titrated into the corresponding buffer solution to exclude the effects resulting from the heat of dilution. The number of titration steps was 25 with a spacing of 250 s for each measurement (25 × 2 μL). After the measurements, the integrated heats of reference titrations (heat of dilution) were subtracted from the integrated heats of adsorption experiments, resulting in normalized heats for each titration step. The normalized heats were then fitted according to an independent binding model^35^ (eq 1) to acquire the association constant (*K*_a_), reaction enthalpy (Δ*H*), and stoichiometry (*n*). This model assumes that a ligand (AB, antibody) binds to one site of a macromolecule (NP, nanoparticle) independently, without cooperative effects.
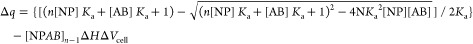
1In eq 1, [NP], [AB], and [NPAB] correspond to the concentrations of the nanoparticles, antibody, and the formed complex, respectively, whereas Δ*V*_cell_ is the change of the total cell volume during the titration. The Gibbs free energy (Δ*G*) is calculated from eq 2 (reaction isotherm equation), whereas *R* and *T* denote the universal gas constant and temperature.
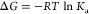
2The change in the entropy (Δ*S*) is determined afterward by eq 3 (Gibbs–Helmholtz equation) after Gibbs free energy (Δ*G*) is calculated.
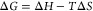
3All results are given as the average of triplicates with the standard deviation that are obtained from three titration experiments for each sample. The data evaluation of the ITC measurements was performed using NanoAnalyze 3.11 software (TA Instruments).

### Cellular Uptake Studies

BT474 cells were seeded into 24-well plates (Greiner Bio-One, Cellstar, Germany) at a density of 100 000 cells per well in 1 mL of Dulbecco’s modified Eagle’s medium (DMEM, Gibco) containing 20% fetal bovine serum (FBS). After overnight incubation at 37 °C in a CO_2_ incubator with 95% humidity and 5% CO_2_ (C2000, Labotect, Germany), the medium was removed. The BT474 cells were then treated with two different concentrations of the antibody-functionalized nanoparticles (75, 300 μg·mL^–1^) at desired time periods (30 min and 2 h). After incubation for the determined time, the medium was removed and washed with 1 mL of PBS. To detach the cells, the cells were trypsinized as per a general procedure that was followed by centrifugation at 500*g* for 5 min. Finally, the obtained cell pellet was resuspended in 1 mL of PBS and measured by flow cytometry.

## Results and Discussion

### Formation and Characterization of Nanoparticles

Carnauba wax nanoparticles were synthesized by utilizing the miniemulsion technique (Figure 1a). The fluorescent tracking molecule, TDI dye, served as a model drug compound, and it was encapsulated into nanoparticles during the formation process. First, the hydrophobic phase containing molten wax, oil ingredients, the drug mimetic (TDI), and an organic solvent (ethyl acetate) was dispersed in the aqueous solution of a nonionic surfactant (Tween 20) by ultrasonication. Second, the dispersion was cooled down to room temperature along with constant stirring and solvent evaporation. This leads to the solidification of the molten wax nanodroplets. Figure 1 shows the colloidal and morphological characteristics of the synthesized carnauba wax nanoparticles. The average sizes of the nanoparticles were about 416 nm with a narrow size distribution range determined by dynamic light scattering measurements (Figure 1a). The nanoparticles were sterically stabilized using a nonionic surfactant, and a slightly negative ζ-potential value was observed due to the fatty acids present in the nanoparticle composition. In addition, the spherical morphology of the particles was confirmed by both TEM and SEM analyses (Figure 1a). As seen in the emission spectra of the nanoparticles (Figure 1b), the encapsulation of the drug mimetic (TDI, *N*,*N*′-(2,6-diisopropylphenyl)-1,6,9,13-tetra[4-(1,1,3,3-tetramethylbutyl)-phenoxy]terrylene-3,4,11,12-tetracarboxidiimide) is successfully achieved. This fluorescent molecule will also help us to track the nanoparticles when evaluating the breast cancer cell uptake in the next part of the study. As a next step, thermal characterization of the nanoparticles was performed using DSC (Figure 1c) to gain more insight into the proposed solidification mechanism during the synthesis of the nanoparticles. The DSC thermogram of the nanoparticles in Figure 1c shows that the melting temperature (*T*_m_ = 80.8 °C) matches well with the carnauba wax presence, whereas the wax–oil particle matrix crystallizes at a temperature of 43.5 °C (*T*_c_). Besides, the broad crystallization range in Figure 1c shows that solidification started before the dispersion reached room temperature during particle formation.

### Antibody Functionalization of Nanoparticles

**Figure 2:**
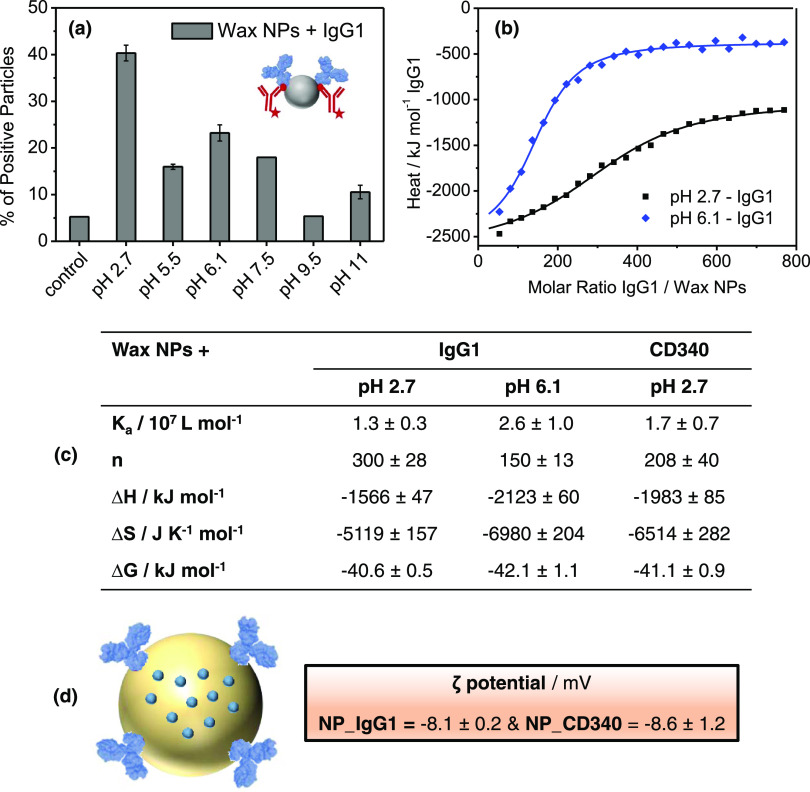
Antibody functionalization of carnauba wax nanoparticles using the pH-dependent adsorption method. (a) Flow cytometry results showing the optimum adsorption at pH 2.7; inset, nanoparticles adsorbed with primary IgG1 antibodies (blue) and secondary Alexa-Fluor 405-labeled antibodies (red) used to detect the primarily adsorbed IgG1. (b) Adsorption isotherms of IgG1 antibodies titrated into the carnauba wax nanoparticles at pH 2.7 (black squares) and pH 6.1 (blue diamonds), *T* = 25 °C, as acquired from isothermal titration calorimetry (ITC) measurements. Isotherms were fitted according to an independent binding model represented by solid lines. (c) Adsorption parameters obtained from ITC measurements by applying the independent binding model. Mean values of triplicates are given with their standard deviations. (d) ζ-Potential values of antibody-decorated carnauba wax nanoparticles.

As a next step, antibody functionalization of the synthesized carnauba wax nanoparticles was performed. The interaction of antibodies and nanoparticles depends on several factors such as the charge, reactive groups, hydrophobicity of the nanoparticle surface together with the antibody subtypes, and the conformational changes at different conditions.^27,32,36^ Therefore, an optimum condition needs to be determined for a specific nanoparticle–antibody system. Since the various pH values can drastically influence the antibody behavior for the adsorption process, we incubated the antibodies with carnauba wax nanoparticles at six different pH conditions. Both IgG1 and CD340 antibodies showed a tendency to adsorb better at acidic pH values, as determined by flow cytometry analysis (Figures 2a and S1). The quantitative results (Figures 2a and S1) indicated that pH 2.7 was the optimum condition for functionalizing the surface of the carnauba wax nanoparticles using both antibodies. This is in agreement with our previous report on antibody adsorption to polystyrene nanoparticles.^27^

We further studied the interaction of antibodies and nanoparticles using isothermal titration calorimetry (ITC) as a complementary method to gain more insight into the binding strength, which is an important parameter for the final nanomedicine performance. We performed this method at pH 2.7 and 6.1 to see whether we could detect the adsorption of antibodies applying ITC under similar conditions of our functionalization approach (Figures 2a,b and S1a,b). The titration of antibodies into nanoparticles at the corresponding pH values resulted in detectable heat changes of the interaction, leading to adsorption isotherms that were fitted according to an independent binding model. To remove the heat of dilution effects, titration of antibodies into the corresponding buffer solution was also performed and subtracted from the heat changes of the main titration before the independent binding fits. It is clearly seen from the adsorption isotherm in Figure 2b that the titration of IgG1 antibodies into the nanoparticle dispersion at both pH 2.7 and pH 6.1 showed an exothermic interaction, which allowed us to calculate the thermodynamic properties (Figure 2c). Similarly, CD340 interaction with nanoparticles at pH 2.7 was detected from the adsorption isotherm, whereas no interaction at the pH 6.1 condition was detectable (Figures 2c and S1). This finding matches well with the flow cytometry results, wherein we have observed the highest adsorption performance of the antibodies at pH 2.7. In addition to that, the second-best adsorption condition was observed at pH 6.1 for IgG1, which is supported by both ITC and flow cytometry experiments. By this, the thermodynamic adsorption parameters between the antibodies and the carnauba wax nanoparticles at corresponding pH values were determined (Figure 2c). The high association constants (*K*_a_) in the range of 10^7^ M^–1^ supported strong binding for both antibodies. The calculated stoichiometry (Figure 2c) for IgG1 at pH 2.7 (*n* = ∼300 ± 28) is two times higher than that at pH 6.1 (*n* = ∼150 ± 13), and this fits well with the flow cytometry results. As seen, a sufficiently enough amount of the CD340 antibody (*n* = 208 ± 40) was adsorbed onto the carnauba wax nanoparticles at pH 2.7 for further breast cancer cell targeting studies. Both of the adsorption isotherms showed exothermic characteristics (Δ*H* < 0), and the nanoparticle–antibody interactions were enthalpy-driven that overcame the unfavorable entropy loss (Δ*S* < 0), as likely seen for most of the protein nanoparticle interactions.^32^

### Targeting BT474 Breast Cancer Cells

**Figure 3:**
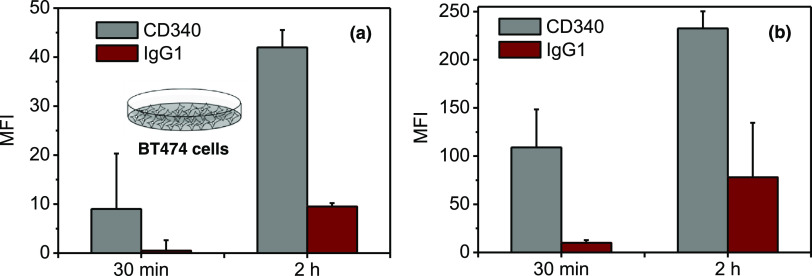
Concentration- and time-dependent uptake of antibody-functionalized carnauba wax nanoparticles (NPs) toward BT474 HER2-positive breast cancer cells. The concentration of NPs is (a) 75 μg·mL^–1^ and (b) 300 μg·mL^–1^. HER2 = human epidermal growth receptor 2, MFI = median fluorescence intensity.

To demonstrate the efficiency of our CD340-decorated carnauba wax nanoparticles for targeting HER2-positive breast cancer cells, the uptake of CD340- and IgG1-functionalized nanoparticles in BT474 cells was assessed using flow cytometry analysis. IgG1 is the isotype control antibody for CD340 that lacks specificity to our target, and it is utilized as a negative control to distinguish the unspecific uptake. BT474 human breast cancer cells are characterized by the overexpression of human epidermal growth factors receptor 2 (HER2).^37,38^Figure 3 shows the flow cytometry histograms of BT474 cells, wherein time- and concentration-dependent uptake of the antibody-functionalized carnauba wax nanoparticles was demonstrated. These results clearly indicate that CD340-modified nanoparticles showed selectivity toward HER2-positive BT474 breast cancer cells at concentrations of both 75 and 300 μg·mL^–1^ at different time spans. At 30 min of cellular uptake and a concentration of 75 μg·mL^–1^, the mean fluorescence intensity (MFI) of the BT474 cells incubated with our negative control, IgG1-modified nanoparticles, was only 0.5, whereas the mean MFI was 9 for CD340-modified nanoparticles (Figure 3a). To enhance the efficiency of uptake, 2 h of incubation time was used. This improves the cellular uptake of CD340-modified nanoparticles of about 5 times, which was still well beyond the uptake of IgG1-modified NPs (Figure 3a). The enhancement in the BT474 cellular uptake of our targeted nanoparticles (CD340-modified) progresses even more at a concentration of 300 μg·mL^–1^ (Figure 3b). In this condition, the CD340_NP uptake was 10 and 13 times higher than that of the cells incubated with IgG1_NPs that lack specificity toward BT474 cells (MFI_ave = 109 at 30 min and 232 at 2 h for CD340_NPs; MFI_ave = 10 at 30 min and 17 at 2 h for IgG1_NPs) (Figure 3b). Such cellular uptake improves because of the successful recognition of the CD340 antibody on the surface of our carnauba wax nanoparticles by the overexpressed HER2 receptors on the BT474 breast cancer cells.

Obviously, the shelf life of our particles from both biological function and physicochemical integrity viewpoints is an important parameter to define the performance of the developed nanoparticles. For this purpose, we also investigated the long-term stability of the antibody-functionalized (both IgG1 and CD340) and naked carnauba wax nanoparticles for 6 months of storage period at 4 °C. First, the physicochemical integrity of the nanoparticles was proved by monitoring the ζ-potential values within the 6 month period (Figure S2). In addition, the nanoparticle morphology after 6 months of storage remained conserved as seen from TEM micrographs (Figure S2). Besides, no phase separation was observed within this time period. After ensuring the physicochemical and morphological stability, the final step was to investigate the biological function of the antibody-functionalized carnauba wax nanoparticles after 6 months of storage period. When we incubate the 6 month-stored CD340- and IgG1-modified nanoparticles (75 μg·mL^–1^) for 2 h with BT474 cells, the uptake of CD340_NPs was about 6.5 fold higher than that of IgG1_NPs (Figure S3). This result confirms that the CD340-decorated carnauba wax nanoparticles show specific cell interaction toward HER2-positive breast cancer cells even after 6 months of storage period. Conservation of the functions for a relatively high time period is a significant success of the developed nanoparticles that enables us to define the limits of the shelf life for complex and costly medical applications.

## Conclusions

In this study, we demonstrated the development of antibody-functionalized carnauba wax nanoparticles and their further use in targeting HER2-positive breast cancer cells. The nanoparticles were efficiently encapsulated with a fluorescent tracking molecule that served as a hydrophobic drug mimetic. The synthesis approach included the formation of molten wax droplets using miniemulsion methods, which in turn were followed by solidifying the nanodroplets by cooling and solvent evaporation, as supported by thermal analysis of the dispersion. The surface of the fabricated nanoparticles was further functionalized with antibodies (targeting antibody = CD340 and isotype control = IgG1) using a pH-dependent adsorption method. Since the adsorption was optimum at acidic conditions acquired from flow cytometry analysis, thermodynamic characteristics of this functionalization process were studied at pH 2.7 and 6.1 using isothermal titration calorimetry (ITC). This concluded that strong binding of antibodies onto the nanoparticle surface can be achieved, as observed from the high association constant values in the range of 10^7^ M^–1^. Besides, the optimum functionalization condition was seen at pH 2.7 for the antibodies (CD340 and IgG1), as determined from both flow cytometry analysis and ITC measurements. This feasible functionalization approach is very useful especially for systems that are devoid of easily accessible chemical reactivity. Thus, we can show a practical and effective surface modification of carnauba wax nanoparticles using pH-dependent adsorption of IgG1 and CD340 antibodies, which provides an alternate method toward complex chemical modification. The described functionalization methodology can be also used to attach various recognition elements to serve for distinct therapies. Successful targeting toward HER2-positive BT474 breast cancer cells using CD340-decorated carnauba wax nanoparticles was another milestone of this work that preserves the ability of specific cellular uptake for the evaluated 6 month period. Overall, we think that inert and nontoxic carnauba wax nanoparticles decorated with recognition units like antibodies have a high potential for use as clinically relevant nanomedicines in drug delivery applications and they can be a safer alternative for various nanoparticle systems in the biomedicine.

## Supporting Information Available

Additional characterization and experimental data (PDF)
