# Supplementary material for: Antibody-Functionalized Carnauba Wax Nanoparticles to Target Breast Cancer Cells
Source: ACS Appl Bio Mater. 2022 Jan 3;5(2):622–9. doi: 10.1021/acsabm.1c01090 (PMC8864612; doi:10.1021/acsabm.1c01090)
Supplement: Supplementary file 1 — mt1c01090_si_001.pdf [file mt1c01090_si_001.pdf]

# Antibody Functionalized Carnauba Wax Nanoparticles to Target Breast Cancer Cells

*Banu Iyisan*[a,b], *Johanna Simon*[a,c], *Yuri Avlasevich*[a], *Stanislav Baluschev*[a,d], *Volker Mailaender*[a,c], *Katharina Landfester*[a,*]

[a]Max Planck Institute for Polymer Research, Ackermannweg 10, 55128 Mainz, Germany

[b]Boğaziçi University, Institute of Biomedical Engineering, 34684 Çengelköy, Istanbul, Turkey

[c]Dermatology Clinic, University Medical Center of the Johannes Gutenberg-University Mainz, Langenbeckstr. 1, 55131 Mainz, Germany

[d]University of Sofia "Saint Kliment Ohridski", Faculty of Physics, James Bourchier 5, 1164 Sofia, Bulgaria

*Address correspondence to landfester@mpip-mainz.mpg.de

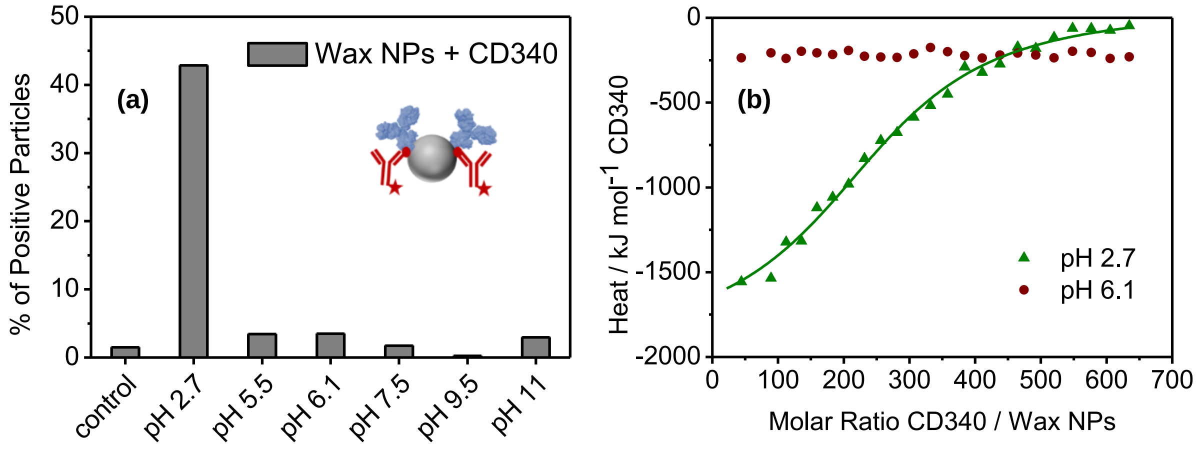


**Figure S1.** Antibody - functionalization of carnauba wax nanoparticles using pH dependent adsorption method. **(a)** Flow cytometry results showing the optimum adsorption at pH 2.7 - inset: nanoparticles adsorbed with primary CD340 antibodies (blue) and secondary Alexa Fluor 405 - labelled antibodies (red) used to detect the primary adsorbed CD340. **(b)** Adsorption isotherms of CD340 antibodies titrated into the carnauba wax nanoparticles at pH 2.7 (green triangles) and pH 6.1 (red circles), $T$ = 25 °C acquired from isothermal titration calorimetry (ITC) measurements. Isotherm at pH 2.7 was fitted according to independent binding model represented by solid lines.

| (a) Stability Check | Wax NP | | Antibody-functionalized Wax NP | |
|---|---|---|---|---|
| | t = 0 | t = 6 months | t = 0 | t = 6 months |
| **Zeta Potential (mV)** | -21.2 ± 0.2 | -21.3 ± 0.5 | -8.1 ± 0.2 | -8.9 ± 0.8 |

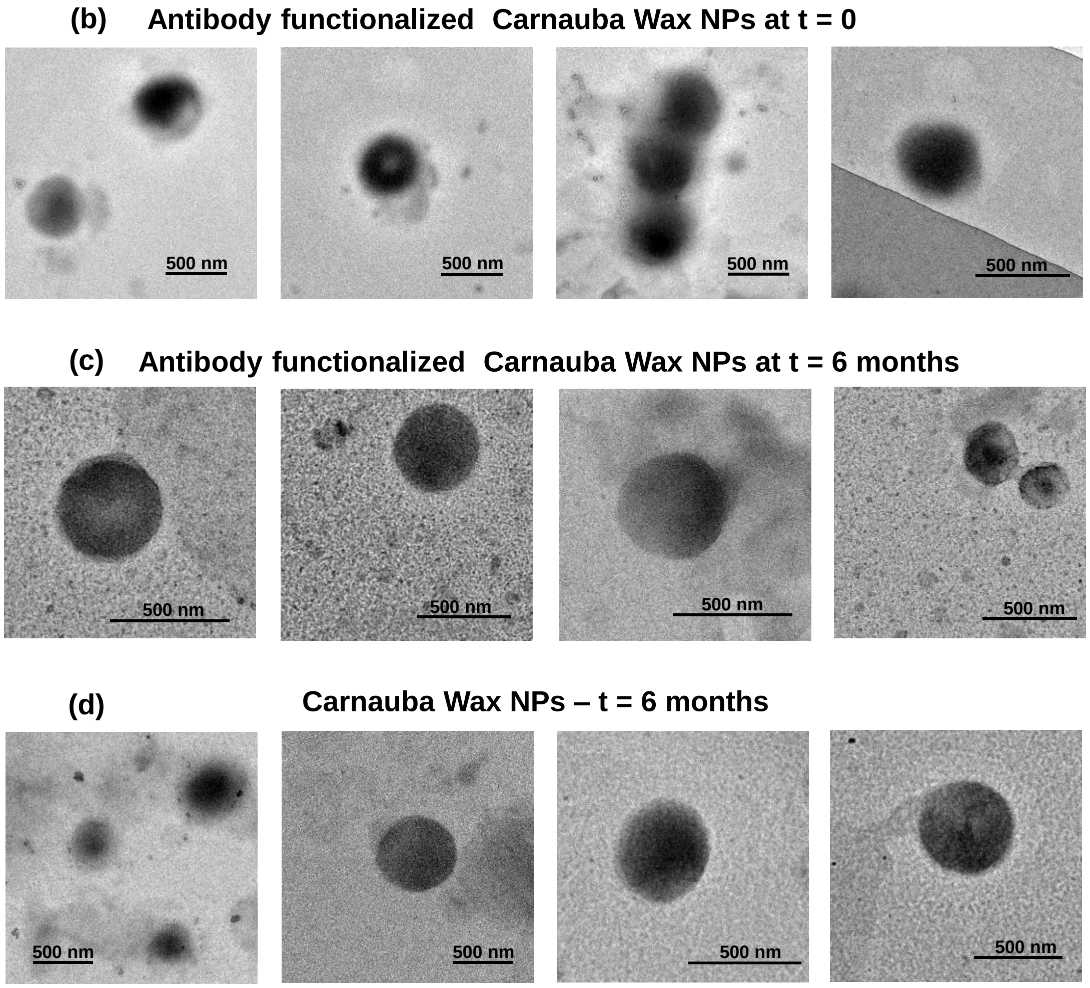


**Figure S2.** Long term physicochemical stability**.** **(a)** Zeta potential values before and after antibody functionalization of carnauba wax nanoparticle proved the integrity within 6 months period of storage at 4 °C. TEM micrographs of antibody functionalized carnauba wax nanoparticles (b) at initial state and (c) after 6 months of storage. (d) TEM micrographs of naked carnauba wax nanoparticles after 6 months of storage period

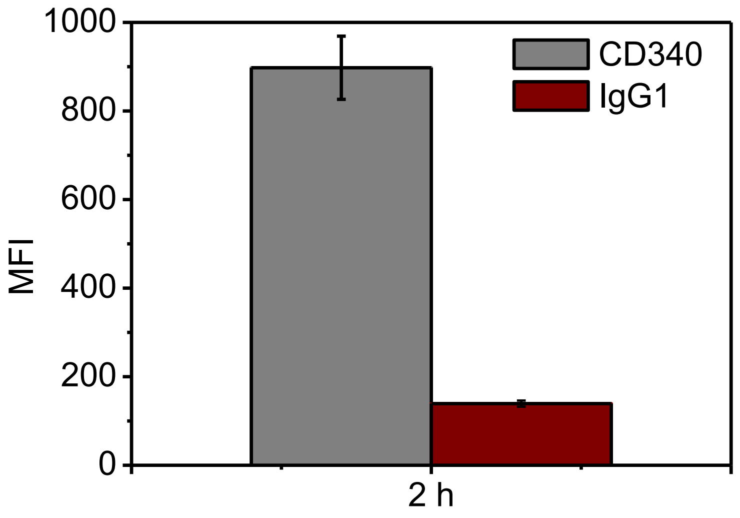


**Figure S3.** Long Term Biological Function**.** Cellular uptake of antibody functionalized carnauba wax nanoparticles towards BT474 HER2-positive breast cancer cells after 6 months storage at 4 °C. Concentration of NPs is 75 $\mu g \cdot mL^{-1}$. HER2 = Human epidermal growth receptor 2, MFI = median fluorescence intensity.

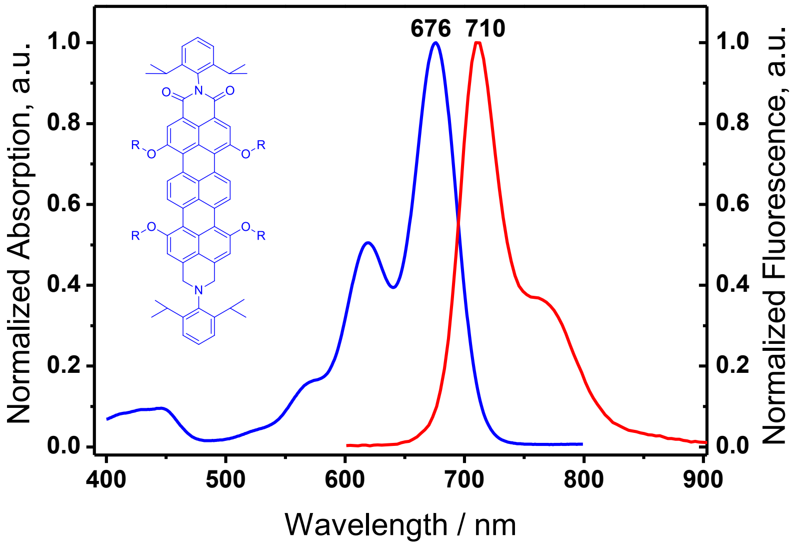


**Figure S4**. Absorption (red lines) and fluorescence emission spectra (blue lines) of tetraphenoxy-TDI (N,N'-(2,6-diisopropylphenyl)-1,6,9,13-tetra[4-(1,1,3,3-tetramethylbutyl)- phenoxy] terrylene-3,4,11,12-tetracarboxidiimide). R= (4-tert-octylphenoxy)

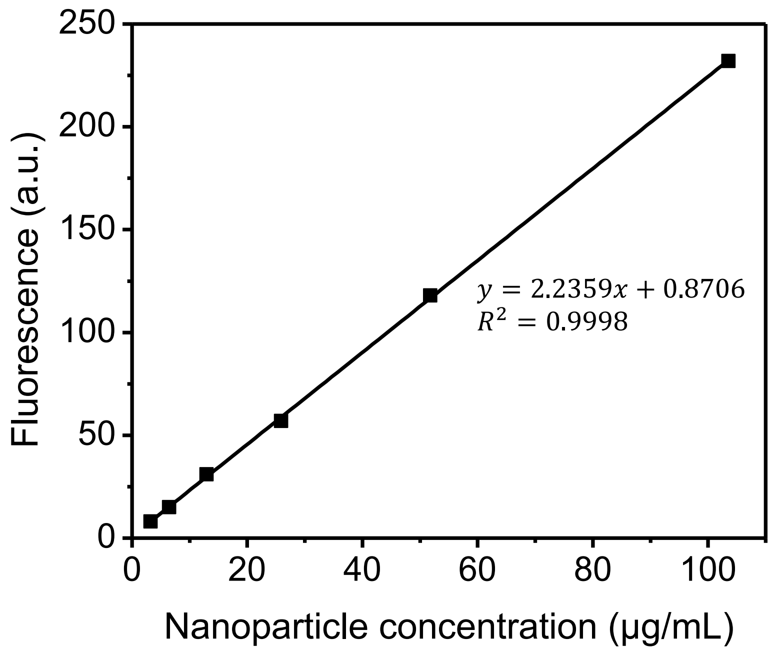


**Figure S5.** Calibration curve used for the determination of the nanoparticle concentration for the antibody-functionalized nanoparticles.

(a)

(b)

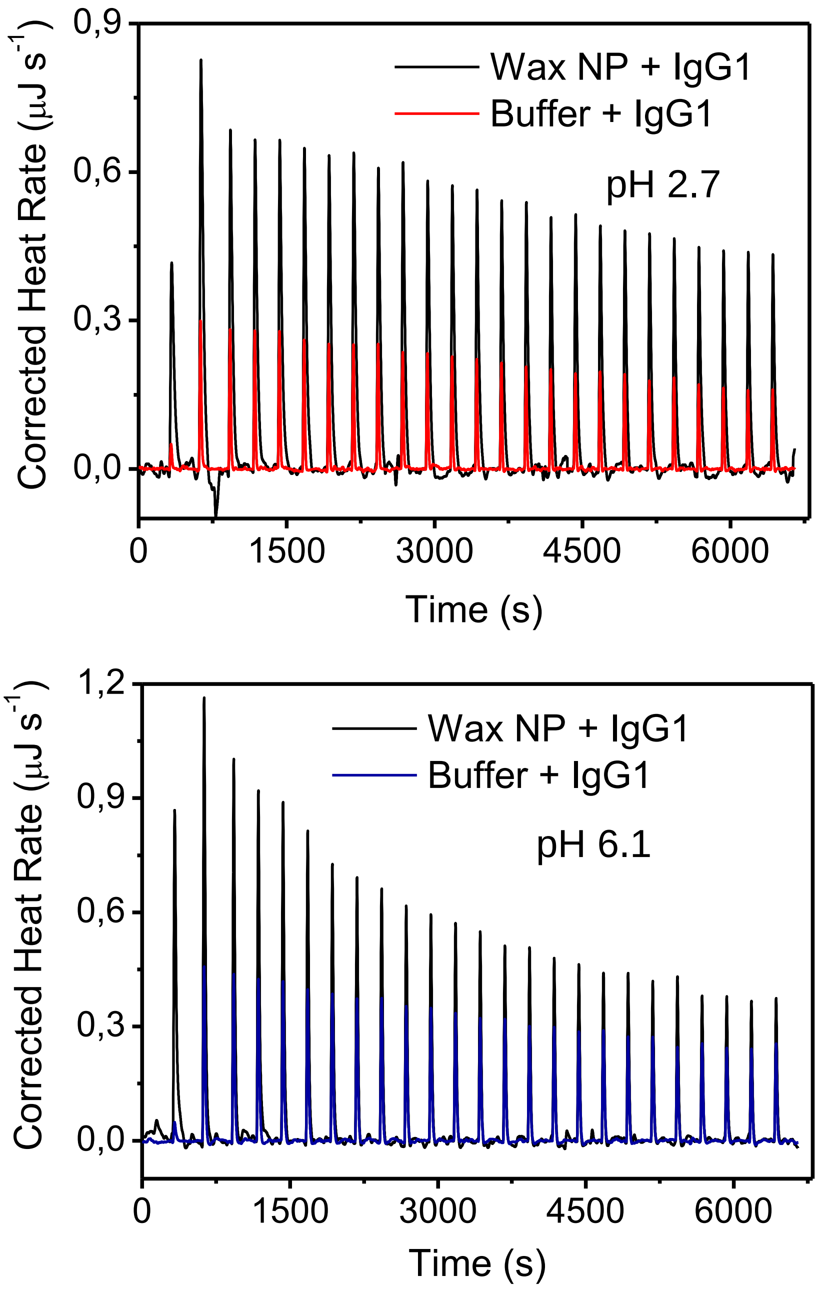


**Figure S6.** ITC raw heat rates for antibody-modification of carnauba wax nanoparticles at **(a)** pH 2.7 and **(b)** at pH 6.1
